# Using discordant twin methods to investigate an environmentally mediated pathway between social support and the reduced likelihood of adolescent psychotic experiences

**DOI:** 10.1017/S0033291719001983

**Published:** 2020-08

**Authors:** Eloise Crush, Louise Arseneault, Andrea Danese, Sara R. Jaffee, Helen L. Fisher

**Affiliations:** 1 King's College London, Social, Genetic & Developmental Psychiatry Centre, Institute of Psychiatry, Psychology & Neuroscience, London, UK; 2 Department of Child & Adolescent Psychiatry, King's College London, Institute of Psychiatry, Psychology & Neuroscience, London, UK; 3 National & Specialist CAMHS Clinic for Trauma, Anxiety, and Depression, South London and Maudsley NHS Foundation Trust, London, UK; 4 Department of Psychology, University of Pennsylvania, Philadelphia, PA, USA

**Keywords:** genetic risk, psychosis, resilience, trauma, twins, victimisation

## Abstract

**Background:**

Social support has been shown to be associated with a reduced likelihood of developing psychotic experiences in the general population and even amongst those at high risk due to exposure to multiple forms of victimisation (poly-victimised). However, it is unclear whether this association is merely due to the confounding effects of shared environmental and genetic influences, or reverse causality. Therefore, we investigated whether social support has a unique environmentally mediated effect on adolescent psychotic experiences after accounting for familial factors, including genetic factors, and also prior psychopathology.

**Methods:**

Participants were from the Environmental Risk (E-Risk) Longitudinal Twin Study, a nationally-representative cohort of 2232 UK-born twins. Adolescents were interviewed at age 18 about psychotic experiences and victimisation exposure since age 12, and their perceptions of social support. Prior childhood mental health problems and psychotic symptoms were assessed at age 12. The discordant twin method was used to disentangle the relative family-wide and unique-environmental effects of social support on psychotic experiences in the general population and among poly-victimised adolescents.

**Results:**

Perceived social support, particularly from friends, was found to have a unique environmentally mediated buffering effect on adolescent psychotic experiences in the whole sample and in the high-risk poly-victimised group.

**Conclusions:**

The protective effects of social support on adolescent psychotic experiences cannot be accounted for by shared environmental or genetic factors, nor by earlier psychopathology. Our findings suggest that early intervention programmes focused on increasing perceptions of social support have the potential to prevent the emergence of psychotic experiences amongst adolescents.

## Introduction

It is widely acknowledged that psychotic experiences such as hearing voices and feeling very paranoid, occur amongst individuals in the general population (McGrath *et al*., 2015). They are relatively common, with prevalence rates around 17% during childhood and 8% during adolescence (Kelleher *et al*., 2012*a*). Poly-victimisation (exposure to two or more types of victimisation) has been found to be a major risk factor for the emergence of psychotic phenomena with the odds of reporting such phenomena around five times higher than in the general population (Arseneault *et al*., 2011; Crush *et al*., 2018*a*). However, a large proportion of children and adolescents in the general population will not develop psychotic phenomena, even in the context of poly-victimisation (Janssen *et al*., 2004; Shevlin *et al*., 2008; Arseneault *et al*., 2011). Research focused on those who do not develop psychotic phenomena, despite being at high risk (poly-victimised), may provide valuable insights into which factors reduce the likelihood of psychotic experiences emerging and thus inform preventive interventions. Preventing early psychotic phenomena is crucial not only because these experiences are extremely distressing for adolescents (Kelleher *et al*., 2015) but also because they have been shown to predict suicidal behaviours (Kelleher *et al*., 2012*b*) and major psychiatric disorders (Fisher *et al*., 2013) in later life.

In a recent paper, we found social support particularly from friends and family to be protective in relation to age-18 psychotic experiences in the general population and amongst a high-risk group of poly-victimised adolescents after controlling for gender, family socioeconomic status, family psychiatric history, and childhood mental health problems including psychotic symptoms (Crush *et al*., 2018*a*). We concluded that these findings provide evidence for an independent protective effect of adolescent social support in relation to psychotic phenomena in a nationally-representative longitudinal cohort study. This paper aims to further interrogate the potentially causal nature of this association by taking advantage of our longitudinal twin sample to control for all unmeasured shared family-wide environmental and genetic factors that could be confounding this association, and also accounting for earlier mental health problems to limit the possibility of reverse causality.

An association which may be causal in nature would be inferred if social support was found to have a direct, environmentally-mediated protective effect on adolescent psychotic phenomena. An environmentally-mediated pathway would suggest that, to some extent, the effect of perceived social support on psychotic phenomena is distinctly environmental in nature. Ultimately, evidence for this would indicate that interventions to increase social support, or perceptions of social support, could be effective in preventing the development of psychotic phenomena in adolescence. In order to investigate this, it is necessary to limit the possibility of alternative explanations, including potential confounding by shared family-wide environmental and genetic factors, and also considering whether reverse causality is operating, all of which are considered herein and discussed below.

There are several non-causal or indirect explanations for why higher levels of social support are associated with a reduced likelihood of developing psychotic experiences during adolescence. Firstly, environments or experiences shared by family members, including the home and community environment could influence perceptions of social support and also protect against the onset of psychotic phenomena. For example, children who grow up in a warm, nurturing home environment are less likely to report psychotic phenomena (Crush *et al*., 2018*b*) and may also perceive others to be more supportive, perhaps due to secure attachment formation (Blain *et al*., 1993). Similarly, individuals who grow up in a neighbourhood with high social cohesion may have more access to social support within their community, while low neighbourhood social cohesion has been found to be associated with the emergence of psychotic phenomena (Newbury *et al*., 2016). Therefore, shared family-wide environmental factors might be confounding the protective effect of social support on psychotic experiences.

Secondly, genetic factors may also explain the association by influencing both perceptions of social support and the propensity to develop psychotic experiences. Indeed, there are modest to high heritability estimates for the emergence of psychotic phenomena during childhood and adolescence (Polanczyk *et al*., 2010; Zavos *et al*., 2014). Additionally, despite intuitively social support being considered an environmental exposure, it is also influenced by genetic factors (Kendler, 1997), with a moderate heritability of 40% found in the current cohort (Matthews *et al*., 2016). Given that social support and psychotic phenomena are both influenced by genetic factors, it is possible that genes may confound the association between them. For example, it is plausible that individuals with paranoia or suspicious thoughts, that may arise from a genetic predisposition towards psychotic experiences, could have problems maintaining relationships with friends (Claes, 1994) and family (Riggio and Kwong, 2011), and be less appealing to new potential friends or partners. Conversely, those without such genetic vulnerability may be more likely to elicit social support and also be protected from developing psychotic phenomena.

Relatedly, the potential for reverse causality should be considered in the association between social support and psychotic phenomena. It is possible that early manifestations of psychosis or other mental health problems in childhood might reduce affected individuals’ social networks, and the resulting social isolation may increase the likelihood of psychotic phenomena developing or persisting. Indeed, individuals with early signs of psychosis have been shown to have limited social networks (Gayer-Anderson and Morgan, 2013), and psychotic symptoms and other mental health problems in childhood have also been shown to increase risk for adolescent psychotic phenomena (Polanczyk *et al*., 2010; Zammit *et al*., 2013). Those without mental health problems in childhood might therefore be more likely to have greater social support and less likely to develop psychotic phenomena in adolescence. Thus, it is important to take into account prior psychopathology to improve understanding of the temporal association between social support and adolescent psychotic phenomena.

This study aims to utilise the discordant twin design (Vitaro *et al*., 2009; Pingault *et al*., 2018) in a longitudinal cohort to consider the relative family-wide *v.* unique environmental effects of social support on adolescent psychotic experiences and control for earlier psychopathology. This approach capitalises on the fact that twins reared together share the same family environment and the same genes (100% for monozygotic [MZ] twins; 50% for dizygotic twins [DZ]). To estimate family-wide effects of social support we will firstly consider between-twin effects, thus testing whether twin pairs with higher social support also have a reduced likelihood of psychotic phenomena relative to other twin pairs with lower levels of social support. Next to estimate the unique environmental effects of social support we will consider the within-twin effects, i.e. whether twins with higher levels of social support than their co-twin also have a reduced likelihood of adolescent psychotic phenomena relative to their co-twin. Modelling these effects together allows us to ascertain the unique environmental effects of social support on psychotic phenomena relative to shared family-wide effects. We will additionally conduct analyses restricted to MZ twins to fully rule out genetic confounding, and also control for psychotic symptoms and other mental health problems in childhood to limit reverse causality. Analyses will be run in the full general population sample and also in the sub-group of adolescents who have been poly-victimised to test whether a unique environmental pathway is still evident in this high-risk group.

## Methods

### Study cohort

Participants were members of the Environmental Risk (E-Risk) Longitudinal Twin Study, which tracks the development of a nationally-representative birth cohort of 2232 British twin children. The sample was drawn from a larger cohort of twins born in England and Wales in 1994–1995 (Trouton *et al*., 2002). Full details about the sample are reported elsewhere (Moffitt and E-Risk Study Team, 2002). Briefly, the E-Risk sample was constructed in 1999–2000, when 1116 families with same-sex 5-year-old twins (93% of those eligible) participated in home-visit assessments. Families were recruited to represent the UK population of families with newborns in the 1990s, based on residential location throughout England and Wales and mothers’ age. Teenaged mothers with twins were over-selected to replace high-risk families who were selectively lost to the register through non-response. Older mothers having twins via assisted reproduction were under-selected to avoid an excess of well-educated older mothers. E-Risk families are representative of UK households across the spectrum of neighbourhood-level deprivation: 25.6% of E-Risk families live in ‘wealthy achiever’ neighbourhoods compared to 25.3% of households nation-wide; 5.3% *v.* 11.6% live in ‘urban prosperity’ neighbourhoods; 29.6% *v.* 26.9% live in ‘comfortably off’ neighbourhoods; 13.4% *v.* 13.9% live in ‘moderate means’ neighbourhoods; and 26.1% *v.* 20.7% live in ‘hard-pressed’ neighbourhoods (Caspi *et al*., 2000; CACI, 2006). E-Risk families under-represent ‘urban prosperity’ neighbourhoods because such households are likely to be childless. The sample comprised 56% MZ and 44% DZ twin pairs, and sex was evenly distributed within zygosity (49% male). All families were English speaking, and the majority (93.7%) were White.

Follow-up home visits were conducted when children were 7 years (98% of the 1116 E-Risk Study families participated), 10 years (96% participation), 12 years (96% participation) and 18 years (93% participation). Home visits at ages 5, 7, 10, and 12 years included assessments with participants as well as their mother (or primary caretaker); the home visit at age 18 included interviews only with the participants. Each twin participant was assessed by a different interviewer. The average age of the 2066 twins at the time of the age 18 assessment was 18.4 years (s.d. = 0.36); all interviews were conducted after the 18^th^ birthday. There were no differences between those who did and did not take part at age 18 in terms of socioeconomic status (SES) assessed when the cohort was initially defined (χ^2^ = 0.86, *p* = 0.65), age-5 IQ scores (*t* = 0.98, *p* = 0.33), or age-5 internalising or externalising behaviour problems (*t* = 0.40, *p* = 0.69 and *t* = 0.41, *p* = 0.68, respectively). The Joint South London and Maudsley and the Institute of Psychiatry Research Ethics Committee approved each phase of the study. Parents gave informed consent, and participants gave assent at ages 5–12 and informed consent at age 18.

### Measures

#### Adolescent psychotic experiences

At age 18, each E-Risk participant was privately interviewed by a trained research worker about 13 psychotic experiences occurring since age 12. Seven items pertained to delusions and hallucinations, such as ‘have you ever thought you were being watched, followed or spied on?’ and ‘do you hear voices that others cannot?’. This interview has been described in detail previously (Polanczyk *et al*., 2010). Interviewers probed responses to these items to ensure that participants understood the questions and obtained descriptions of their experiences. The other six items pertained to unusual experiences which drew on item pools since formalised in prodromal psychosis instruments including the PRIME-screen and SIPS (Loewy *et al*., 2011). These included ‘I worry that my food may be poisoned’ and ‘My thinking is unusual or frightening’. Further information on this measure is provided in the online Supplementary Materials. Interviewers coded each item 0, 1, 2 indicating respectively ‘not present’, ‘probably present’, and ‘definitely present’. All 13 items were summed to create a psychotic experiences scale (range = 0–18, *M* = 1.19, s.d. = 2.58). Just over 30% of participants had at least one psychotic experience between ages 12 and 18 (*n* = 623, 30.2%). This is similar to the prevalence of self-reported psychotic experiences in other community samples of teenagers and young adults (Yoshizumi *et al*., 2004; Kelleher *et al*., 2012*c*). Almost all of the twins who participated in the age-18 assessment reported on their psychotic experiences (*N* = 2063, 99.9%). An overview of the demographics of the sample with psychotic experiences data available is provided in Table 1.

**Table 1.** Demographic characteristics of the Environmental Risk study cohort with psychotic experiences data available at age 18

Characteristic	Whole sample (*N* = 2063) *n* (%)
Gender	
Male	980 (47.5)
Female	1083 (52.5)
Zygosity	
Monozygotic	1164 (56.4)
Dizygotic	899 (43.6)
Family socioeconomic status	
Low	691 (33.5)
Intermediate	683 (33.1)
High	689 (33.4)
	Mean (standard deviation)
Age at interview	18.4 years (0.36)

#### Social support

Social support was assessed using the Multidimensional Scale of Perceived Social Support (MSPSS), which assesses individuals’ access to supportive relationships with family, friends and significant others (Zimet *et al*., 1988). The 12 items in the MSPSS consist of statements such as ‘There is a special person who is around when I am in need’ and ‘I can count on my friends when things go wrong’. At age 18, participants rated these statements as ‘not true’ (0), ‘somewhat true’ (1), or ‘very true’ (2). We summed the scores to produce an overall social support scale with higher scores reflecting greater social support (internal consistency: *α* = 0.88). In addition, each of the three sub-scales (social support from either family, friends or significant others) were utilised separately to examine whether the findings were consistent across these different sources of social support.

#### Adolescent poly-victimisation

At age 18, participants were interviewed about exposure to a range of adverse experiences between 12–18 years using the Juvenile Victimization Questionnaire, 2^nd^ revision (JVQ-R2) (Finkelhor *et al*., 2011) adapted as a clinical interview, which has been outlined in a previous paper (Fisher *et al*., 2015) and in the online Supplementary Materials. Each twin was interviewed by a different research worker, and each JVQ question was asked for the period ‘since you were 12’. Age 12 is a salient age for our participants because it is the age when British children leave primary school to enter secondary school. Our adapted JVQ comprised 45 questions covering 7 different forms of victimisation: maltreatment, neglect, sexual victimisation, family violence, peer/sibling victimisation, internet/mobile phone victimisation, and crime victimisation. The worst experience (according to the participant) for each victimisation type was rated by trained coders using a 6-point scale: 0 = not exposed, then 1–5 for increasing levels of severity. The adolescent poly-victimisation variable was derived by summing all victimisation experiences that received a code of ‘4’ or ‘5’ (i.e. severe exposure). Due to small numbers in some of the groups, we collapsed this variable into ‘0’ not victimised (64.6%), ‘1’ experienced 1 type of severe victimisation (19.2%), and ‘2’ poly-victimised (16.2%; experienced 2 or more types of severe victimisation).

#### Potential confounders

Family SES was measured via a composite of parental income (total household), education (highest for mother/father), and occupation (highest for mother/father) when children were aged 5 (Trzesniewski *et al*., 2006), and was categorised into tertiles (i.e. low-, medium-, and high-SES). Childhood psychotic symptoms pertaining to seven delusions and hallucinations were measured when children were aged 12 during private interviews. Items and interviewer notes were assessed by a psychiatrist expert in schizophrenia, a psychologist expert in interviewing children, and a child and adolescent psychiatrist to verify the validity of the symptoms (Polanczyk *et al*., 2010). A total of 5.9% of children reported experiencing at least one definite psychotic symptom at age 12 (*N* = 125). In order to capture earlier psychopathology more broadly, a variable was also created for the presence *v.* absence of any childhood mental health problems that captured children who met criteria for extreme anxiety, clinically-relevant depression symptoms, attention deficit hyperactivity disorder (ADHD), or conduct disorder by age 12 (see online Supplementary Materials).

### Statistical methods

Analyses were conducted in STATA 15 (Stata-Corp, College Station, TX). We applied Generalised Estimating Equations (GEE) with binominal function specified (logistic regression) and an exchangeable correlation structure to account for familial clustering in order to simultaneously estimate the family-wide (between-twin pair) and unique (within-twin pair) effects of social support on age-18 psychotic experiences (Carlin *et al*., 2005; Vitaro *et al*., 2009). The between-twin pair analysis considers whether pairs of twins with higher social support than other twin pairs are also less likely to have psychotic experiences. In contrast, the within twin-pair analysis considers whether the twin with higher social support than his or her co-twin is also less likely to have psychotic experiences than his or her co-twin (Carlin *et al*., 2005). Because co-twins share their rearing environment as well as half (dizygotic twins) or all (monozygotic twins) their genes, significant *within-twin pair effects* would indicate that social support is associated with a reduced likelihood of psychotic experiences independent of latent, family-wide factors, thus suggesting a unique environmental effect of social support. Further restricting analyses to MZ twins fully rules out genetic influences, and additionally controlling for age-12 psychotic symptoms and other childhood mental health problems accounts for the possibility of reverse causality. All analyses were conducted in the full general population sample and also in the sub-group of poly-victimised adolescents where both twins were poly-victimised (*n* = 158).

## Results

We have previously shown that social support was associated with a reduced likelihood of age-18 psychotic experiences amongst adolescents in the whole sample (OR = 0.91, 95% CI 0.89–0.93, *p* < 0.001) and amongst a high-risk group exposed to poly-victimisation (OR = 0.93; 95% CI 0.88–0.98, *p* = 0.009) after controlling for gender, family SES, age-12 psychotic symptoms and other mental health problems at age 12 (Crush *et al*., 2018*a*). There was a reasonable level of discordance among both MZ (30.2%) and DZ (32.7%) twin pairs for age-18 psychotic experiences, and the intraclass correlation for the total social support score at age 18 was greater than 0 but less than 1 for both MZ (ICC = 0.46, 95% CI 0.40–0.52) and DZ (ICC = 0.15, 95% CI 0.06–0.24) twins indicating that there was both within- and between-twin pair variation in these measures.

### Is the association between increased social support and a reduced likelihood of psychotic experiences during adolescence accounted for by shared environmental and genetic factors?

Using discordant twin analyses in the whole sample, we considered the association between overall social support, and each sub-type of social support, with a reduced likelihood of adolescent psychotic experiences at age 18. We found that these associations were explained by family-wide effects of social support shared by twin pairs (Table 2), therefore showing that twin pairs with higher social support were less likely to report psychotic experiences relative to twin pairs who reported lower social support. Notably, we also found evidence of a unique environmental effect, whereby higher perceived social support by one twin relative to their co-twin was associated with a reduced likelihood of psychotic experiences (Table 2). When analyses were repeated for MZ twins only (to fully control for genetic influences), we again found both family-wide influences and the unique environment to be implicated in the associations for total social support and for support from both family and friends (Table 2), although the findings were inconclusive in relation to social support from significant others. The fact that the results showed a significant within-twin-pair association for total social support, and also social support from friends and family, with a reduced likelihood of psychotic experiences in the whole sample and MZ group provides support for the presence of a unique environmentally-mediated protective pathway for these social support types.

**Table 2.** Family-wide and unique environmental effects of social support on age-18 psychotic experiences

Social support scale	Whole sample				Both twins poly-victimised			
	All twins *N* = 2058		MZ twins *N* = 1162		All twins *N* = 158		MZ twins *N* = 96	
	Adjusted OR^a^ (95% CI)	*p* Value	Adjusted OR^a^ (95% CI)	*p* Value	Adjusted OR^a^ (95% CI)	*p* Value	Adjusted OR^a^ (95% CI)	*p* Value
Total								
Family-wide	**0.89 (0.86–0.92)**	<0.001	**0.91 (0.88–0.95)**	<0.001	0.94 (0.84–1.04)	0.237	0.98 (0.85–1.11)	0.710
Unique	**0.93 (0.90–0.96)**	<0.001	**0.93 (0.89–0.97)**	<0.001	0.91 (0.82–1.00)	0.052	0.92 (0.81–1.04)	0.193
Family								
Family-wide	**0.75 (0.70–0.80)**	<0.001	**0.77 (0.70–0.84)**	<0.001	0.85 (0.70–1.02)	0.077	0.84 (0.66–1.06)	0.143
Unique	**0.85 (0.78–0.92)**	<0.001	**0.86 (0.77–0.96)**	0.007	**0.81 (0.61–1.08)**	0.140	0.80 (0.61–1.05)	0.114
Friends								
Family-wide	**0.78 (0.73–0.84)**	<0.001	**0.82 (0.76–0.90)**	<0.001	0.90 (0.73–1.10)	0.303	1.03 (0.80–1.33)	0.831
Unique	**0.88 (0.82–0.94)**	<0.001	**0.88 (0.80–0.96)**	0.004	0.81 (0.69–0.96)	0.012	0.85 (0.69–1.04)	0.121
Significant Others								
Family-wide	**0.90 (0.84–0.97)**	0.004	0.97 (0.89–1.07)	0.535	1.01 (0.79–1.30)	0.914	1.15 (0.87–1.51)	0.321
Unique	**0.92 (0.86–0.99)**	0.023	0.92 (0.84–1.02)	0.120	0.92 (0.68–1.24)	0.592	1.00 (0.62–1.63)	0.984

CI, confidence interval; MZ, monozygotic; OR, odds ratio.

Bold text indicates *p* < 0.05. Family-wide indicates between–twin pair difference; unique, within–twin pair difference.

a Adjusted for child's gender.

Similarly, amongst twins exposed to poly-victimisation, we found evidence of a unique environmental pathway between social support from friends and a reduced likelihood of adolescent psychotic experiences (Table 2). When analyses were restricted to MZ twins only who had both been exposed to poly-victimisation, we found similar trends (Table 2). However, these effects were not significant, possibly due to the relatively small number of twins (*N* = 96) within this sub-group.

### Is the association between increased social support and a reduced likelihood of psychotic experiences accounted for by childhood psychopathology?

Next, in order to investigate any potential reverse causality between social support and psychotic experiences, we controlled for psychotic symptoms and other mental health problems at age 12 to support the interpretation of the directionality of the association. We found that the unique environmental effect of total social support on the reduced likelihood of adolescent psychotic experiences remained significant when accounting for earlier psychopathology within the full sample and when analyses were restricted to MZ twins (Table 3), thus suggesting this type of reverse causality was unlikely to be operating. The buffering effects of support from friends and family were also robust to adjustment for childhood psychopathology and shared family factors (Table 3).

**Table 3.** Family-wide and unique environmental effects of social support on age-18 psychotic experiences controlling for childhood mental health problems

Social support scale	Whole sample				Both twins poly-victimised			
	All twins *N* = 2058		MZ twins *N* = 1162		All twins *N* = 158		MZ twins *N* = 96	
	Adjusted OR^a^ (95% CI)	*p* Value	Adjusted OR^a^ (95% CI)	*p* Value	Adjusted OR^a^ (95% CI)	*p* Value	Adjusted OR^a^ (95% CI)	*p* Value
Total								
Family-wide	**0.90 (0.87–0.93)**	<0.001	**0.92 (0.88–0.96)**	<0.001	0.93 (0.83–1.04)	0.191	0.97 (0.83–1.13)	0.674
Unique	**0.93 (0.90–0.96)**	<0.001	**0.92 (0.88–0.97)**	0.001	**0.90 (0.82–1.00)**	0.045	0.91 (0.80–1.04)	0.163
Family								
Family-wide	**0.77 (0.72–0.83)**	<0.001	**0.79 (0.72–0.87)**	<0.001	0.83 (0.68–1.01)	0.058	0.80 (0.63–1.03)	0.083
Unique	**0.86 (0.79–0.93)**	<0.001	**0.86 (0.76–0.97)**	0.012	0.85 (0.64–1.13)	0.256	0.81 (0.60–1.08)	0.144
Friends								
Family-wide	**0.80 (0.75–0.85)**	<0.001	**0.84 (0.77–0.91)**	<0.001	0.91 (0.74–1.11)	0.339	1.08 (0.84–1.39)	0.531
Unique	**0.87 (0.81–0.93)**	<0.001	**0.86 (0.78–0.96)**	0.004	**0.78 (0.66–0.92)**	0.003	0.82 (0.65–1.03)	0.092
Significant Others								
Family-wide	**0.91 (0.85–0.98)**	0.011	0.97 (0.88–1.07)	0.596	0.99 (0.74–1.32)	0.928	1.13 (0.79–1.64)	0.502
Unique	**0.92 (0.85–0.99)**	0.020	0.91 (0.82–1.01)	0.092	0.89 (0.65–1.20)	0.440	1.00 (0.59–1.67)	0.989

CI, confidence interval; MZ, monozygotic; OR, odds ratio.

Bold text indicates *p* < 0.05. Family-wide indicates between–twin pair difference; unique, within–twin pair difference.

a Adjusted for child's gender, age-12 psychotic symptoms, and other mental health problems at age 12.

A similar pattern of results was found after controlling for earlier mental health problems in the poly-victimised group, with total social support and support from friends continuing to have a significant unique environmental effect on the reduced likelihood of adolescent psychotic experiences (Table 3). When restricting analyses to MZ twins, only non-significant trends were found (Table 3) but again this was probably due to the small number in this high-risk sub-group (*N* = 96).

## Discussion

This is the first study to consider whether social support has an environmentally-mediated effect on psychotic phenomena amongst adolescents in the general population and amongst those at high risk by virtue of having been exposed to poly-victimisation. Our findings indicate a unique environmental buffering effect of higher perceived social support on psychotic phenomena amongst our general population sample. Whilst it is important to note that effects sizes were relatively modest in size, these effects were apparent in relation to overall social support and also separately for social support from friends and family. These results held after accounting for shared family-wide environmental and genetic influences as well as for earlier psychopathology thus limiting these potentially confounding effects and reverse causality explanations. We also found evidence for a direct environmentally-mediated protective effect of perceived social support on adolescent psychotic experiences even within the high-risk poly-victimised group. Collectively, these findings provide initial evidence for a possible causal association between higher perceived social support and the reduced likelihood of adolescent psychotic experiences and therefore add weight to the importance of social support as a potential candidate for preventive interventions focused upon adolescent psychotic phenomena.

No prior research has addressed this particular question and thus it is not possible to draw comparisons directly with previous research. Nonetheless, one study conducted in adults diagnosed with psychotic disorders reported a similar buffering effect of social support following exposure to specific types of childhood victimisation (Gayer-Anderson *et al*., 2015). Moreover, in this cohort we have previously reported that warm parent-child relationships had an environmentally mediated protective effect on children's behavioural adjustment following bullying victimisation (Bowes *et al*., 2010). Although these findings relate to a different type of psychopathology, they suggest that supportive relationships may promote resilience to a range of mental health outcomes following victimisation exposure in childhood. Given the paucity of research in this area, more studies are needed and our findings require replication in other large population-based cohorts.

Overall our findings have practical implications as they suggest that early prevention efforts focusing upon improving perceived social support – through greater availability of supportive figures or enhancing perceptions of existing social support, could be effective in protecting against the development of psychotic phenomena in adolescence. Interventions aimed at improving social support from family and peers have previously been found to be effective amongst individuals who already have psychosis (Pilling *et al*., 2002; Norman *et al*., 2005; Castelein *et al*., 2008). Whilst family interventions have been most widely applied, recently there has been increased support for the use of peer interventions (Harrop *et al*., 2015; Morin *et al*., 2017) which our findings also indicate might be helpful. Given resources for interventions are limited, it is possible that internet-based peer support networks could represent a promising solution (Álvarez-Jiménez *et al*., 2012; Naslund *et al*., 2016). Our findings support targeting perceptions of social support amongst adolescents exposed to poly-victimisation. This is practically relevant for clinicians given it has been suggested that individuals exposed to complex trauma may need specific types of treatment or interventions compared to those not exposed (Cook *et al*., 2005).

There are several mechanisms through which social support may exert protective influences in relation to psychotic experiences in both the general population and amongst poly-victimised individuals. For instance, it is possible that social support may facilitate stress reduction (Cohen and Wills, 1985), improve self-esteem (Dumont and Provost, 1999; Turner *et al*., 2015), and may also reduce feelings of loneliness (Sündermann *et al*., 2014), which have all been implicated in the development of psychotic phenomena (Corcoran *et al*., 2003; Smith *et al*., 2006; Pruessner *et al*., 2011; Lim *et al*., 2018).

### Limitations

Some limitations warrant consideration. Firstly, both social support and psychotic experiences were measured at age 18 which has implications for interpreting the directionality of the association between them. We did, however, control for a broad range of earlier mental health problems at age 12, including age-12 psychotic symptoms, to account for this as far as possible and are thus able to largely rule out reverse causality. Research around the consistency of social support during adolescence has not suggested any fundamental shifts in total social support levels over time, whilst there are trends for family support being replaced by peer support, relatively stable levels of support appear to be maintained (Levitt *et al*., 1993; Cantin and Boivin, 2004). Relatedly, as the social support scale used is a subjective measure reflecting individuals’ perceptions of support from friends, family and significant others, it is possible that adolescents who develop psychotic experiences perceive their social support levels to be lower than they actually are. Therefore, we welcome replication of our findings amongst cohorts with co-informant measures of social support. Additionally, due to the number of poly-victimised adolescents being relatively low (*N* = 334), our ability to detect some associations may have been affected by this. Moreover, we defined poly-victimisation as reported exposure to two or more severe types of victimisation in adolescence which, although it is a commonly used approach, means individuals in this group may have experienced quite different combinations of victimisation. Other approaches, such as latent class analysis, have been used to create more homogeneous groupings of victimisation types (e.g. Ford *et al*., 2010), and it is possible that utilisation of such methods might have led to different findings. Our analyses thus warrant replication in even larger twin cohorts and with different methods of conceptualising poly-victimisation. Finally, our measure of psychotic experiences was a self-report measure and it is possible that it captured some genuine experiences (e.g. being followed by someone), as well as psychotic phenomena (e.g. being followed by a fictional character). Nonetheless, we have previously shown that higher levels of social support were associated with a reduced likelihood of clinically-verified adolescent psychotic symptoms (Crush *et al*., 2018*a*), indicating that this is unlikely to have substantially affected our findings.

## Conclusion

The association between greater perceived social support and a reduced likelihood of psychotic experiences in adolescence appears to be extremely robust, even in the context of poly-victimisation, as it was not fully accounted for by family-wide environmental or genetic factors nor was there any evidence of reverse causation. These findings suggest that early intervention programmes focused on increasing perceptions of social support, particularly from friends, have the potential to prevent the emergence of psychotic experiences amongst adolescents. Given that there are finite resources for interventions, efforts might be most efficiently targeted at adolescents exposed to multiple forms of victimisation who are at high risk of developing psychotic phenomena.

## Supplementary material

For supplementary material accompanying this paper visit https://doi.org/10.1017/S0033291719001983. click here to view supplementary material
