# Supplementary material for: Using discordant twin methods to investigate an environmentally mediated pathway between social support and the reduced likelihood of adolescent psychotic experiences
Source: Psychol Med. 2019 Aug 15;50(11):1898–905. doi: 10.1017/S0033291719001983 (PMC7477362; doi:10.1017/S0033291719001983)
Supplement: Supplementary file 1 [file S0033291719001983sup001.doc]

**Supplementary Materials**

**Assessment of victimisation in adolescence**

We have previously reported evidence on the reliability and validity of our measurement of adolescent victimisation.^1^ Here we summarise the method. Participants were interviewed about experiences between 12-18 years using the Juvenile Victimization Questionnaire (JVQ),^2,3^ adapted as a clinical interview. The JVQ has good psychometric properties^4^ and was used in the U.K. National Society for the Prevention of Cruelty to Children national survey,^5,6^ thereby providing benchmark values for comparisons with our cohort.

Within each pair of twins in our cohort, co-twins were interviewed separately by a different research worker and were assured of the confidentiality of their responses. The participants were advised that confidentiality would only be broken if they told the research worker that they were in immediate danger of being hurt, and in such situations the project leader would be informed and would contact the participant to discuss a plan for safety. We assessed 7 different forms of victimisation: maltreatment, neglect, sexual victimisation, family violence, peer/sibling victimisation, internet/mobile phone victimisation, and crime victimisation. Each JVQ question was asked for the period ‘since you were 12’. Participants were given the option to say “yes” or “no” as to whether each type of victimisation had occurred in the reporting period. Research workers could rate each item “maybe” if the participant seemed unsure or hesitant in their response or they were not convinced that the participant understood the question or was paying attention. Items rated as “maybe” were recoded as “no” or “yes” by the rating team based on the notes provided by the research workers. When insufficient notes were available, these responses were recoded conservatively as a “no”. Consistent with the JVQ manual,^2,3^ participants were coded as 1 if they reported any experience within each type of victimisation category, or 0 if none of the experiences within the category were endorsed. If an experience was endorsed within a victimisation category, follow-up questions were asked concerning how old the participant was when it (first) happened, whether the participant was physically injured in the event, whether the participant was upset or distressed by the event; and how long it went on for (by marking the number of years on a Life History Calendar.^7^ In addition, the interviewer wrote detailed notes based on the participant’s description of the worst event. If multiple experiences were endorsed within a victimisation category, the participant was asked to identify and report about their worst experience.

All information from the JVQ interview was compiled into victimisation dossiers. Using these dossiers, each of the seven victimisation categories was rated by an expert in victimology and 3 other members of the E-Risk team who were trained on using the rating criteria. Ratings were made using a 6-point scale: 0 = not exposed, then 1-5 for increasing levels of severity. The anchor points for these ratings were adapted from the coding system used for the Childhood Experience of Care and Abuse interview (CECA),^8,9^ which has good inter-rater reliability.^8,10^ The CECA is a comprehensive semi-structured interview whose standardized coding system attempts to improve the objectivity of ratings by basing them on the coder’s perspective (rather than relying on the participant’s judgment) and focusing on concrete descriptions rather than perceptions or emotional responses to the questions, together with considering the context in which the adverse experience occurred.

In our adapted coding scheme, the anchor points of the scale differ for each victimisation category, with some focused more on the severity of physical injury that is likely to have been incurred during victimisation exposure (crime victimisation, family violence, maltreatment), while others are more focused on the frequency of occurrence of victimisation (peer/sibling victimisation and internet/mobile phone victimisation), the physical intrusiveness of the event (sexual victimisation), or the pervasiveness of the effects of victimisation (neglect). This reflects the different ways in which severity has previously been defined for different types of victimisation.^8,11^ (Given that our sample comprises twins, we also coded if any of the victimisation events experienced by each twin had been perpetrated by their co-twin, as it is possible that growing up with a genetically related, same-age child could increase or decrease sibling victimisation rates.) Each twin’s dossier was evaluated separately, and we did not use information provided in the co-twin’s dossier about their own or shared victimisation experiences to rate direct or witnessed violence exposure for the target twin. High levels of inter-rater reliability were achieved for the severity ratings for all forms of victimisation: crime victimisation (intra-class correlation coefficient [ICC] = 0.89, p < 0.001), peer/sibling victimisation (ICC = 0.91, p < 0.001), internet/mobile phone victimisation (ICC = 0.90, p < 0.001), sexual victimisation (ICC = 0.87, p < 0.001), family violence (ICC = 0.93, p < 0.001), maltreatment (ICC = 0.90, p < 0.001), and neglect (ICC = 0.74, p < 0.001).

The ratings for each type of victimisation were then grouped into three classes: 0 – no exposure (score of 0), 1 – some exposure (score of 1, 2 or 3), and 2 – severe exposure (score of 4 or 5) due to small numbers for some of the rating points. Combining ratings of 4 and 5 is also consistent with previous studies using the CECA, which have collapsed comparable scale values to indicate presence of ‘severe’ abuse.^8,10,12,13^ The adolescent poly-victimisation variable was derived by summing all victimisation experiences that received a code of ‘4’ or ‘5’ (i.e., severe exposure): 64.6% of adolescents had zero severe victimisation experiences; 19.2% had 1; 9.4% had 2; 4.5% had 3; 1.5% had 4; 0.5% had 5; and 0.2% had 6 severe victimisation experiences. Due to small numbers in some of the groups, we collapsed this variable into ‘0’ not victimised, ‘1’ experienced 1 type of severe victimisation, and ‘2’ poly-victimised (experienced 2 or more types of severe victimisation).

**Measure of childhood mental health problems**

A variable for childhood mental health problems was derived to capture children who met criteria for extreme anxiety, clinically-relevant depression symptoms, attention deficit hyperactivity disorder (ADHD), or conduct disorder by age 12. Anxiety was assessed when children were aged 12, via private interviews using the 10-item version of the Multidimensional Anxiety Scale for Children (MASC).^14^ An extreme anxiety group was formed with children who scored at or above the 95th percentile (N = 129, 6.1%). Depression symptoms were assessed at age 12 using the Children’s Depression Inventory (CDI).^15^ Children who scored 20 or more were deemed to have clinically significant depressive symptoms (N = 74, 3.5%). ADHD was assessed using the DSM-IV and the requirement of symptom onset prior to age 12 was met if parents or teachers reported more than 2 ADHD symptoms at ages 5, 7, 10, or 12 years. We derived diagnoses of conduct disorder on the basis of mothers’ and teachers’ reports of children’s behaviour problems using the Achenbach family of instruments and additional DSM-IV items assessing conduct disorder which have previously been described.^16^ Conduct disorder was assumed present if it was diagnosed at ages 5, 7, 10 or 12 years. This variable was dichotomised to distinguish between the presence of any of the above mental health problems (coded 1) versus the absence of any age 12 mental health problems (coded as 0).

**Measure of adolescent psychotic experiences**

To measure adolescent psychotic experiences, E-Risk families were visited by mental health trainees or professionals when children were aged 18. Interviewers had no prior knowledge about the participant. Each participant was privately interviewed about thirteen psychotic experiences they may have experienced since the age of 12, including seven items pertaining to hallucinations and delusions, such as: Have other people ever read your thoughts? Have you ever believed that you were being sent special messages through the television or radio, or that a programme has been arranged just for you alone? Have you ever thought you were being followed or spied on? Have you ever heard voices that other people cannot hear? Have you ever felt like you were under the control of some special power? Have you ever known what another person was thinking, like you could read their mind? Have you ever seen something or someone that other people could not see?; as well as six items about unusual feelings and thoughts such as: I have become more sensitive to lights or sounds, I feel as though I can’t trust anyone, I worry that my food may be poisoned, People or places I know seem different, I believe I have special abilities or powers beyond my natural talents, and My thinking is unusual or frightening. The item choice was guided by the Dunedin Study's age-11 interview protocol,^17^ an instrument prepared for the Avon Longitudinal Study of Parents and Children,^18^ and item pools since formalised in prodromal psychosis screening instruments including the Prevention through Risk Identification, Management and Education (PRIME)-screen,^19^ and the Structured Interview for Psychosis-Risk Syndromes (SIPS).^20^ Interviewers coded each of the 13 items (7 hallucination/delusion items plus 6 unusual experiences items) 0, 1, 2, indicating respectively “not present”, “probably present” and “definitely present”. Responses to each of the 13 items (none, probable, definite) were summed to create a psychotic experiences scale (potential range=0–26, actual range=0–18, M=1.19, SD=2.58). The psychotic experiences measure did not involve clinical verification, meaning that this is a self-report measure capturing a broader range of mild, moderate and potentially clinically pertinent hallucinations, delusions, and other unusual feelings and thoughts. Since there were low numbers of adolescents with high psychotic experiences scores (e.g., only 1.0% [N=21] of participants had a psychotic experiences score of 13 or more), scores were placed into an ordinal scale to tackle the skewed distribution while retaining more information than a binary score. Just over 30% of participants had at least one psychotic experience between ages 12 and 18: 69.8% reported no psychotic experiences (coded 0; N=1,440), 15.5% reported 1 or 2 psychotic experiences (coded 1; N=319), 8.1% reported 3–5 psychotic experiences (coded 2: N=166), and 6.7% reported 6 or more psychotic experiences (coded 3: N=138). This 30.2% prevalence is similar to the prevalence of self-reported psychotic experiences in other community samples of teenagers and young adults.^21-23^

**References**

1. Fisher HL, Caspi A, Moffitt TE, et al. Measuring adolescents’ exposure to victimisation: The Environmental Risk (E-Risk) Longitudinal Twin Study. *Dev Psychopathol.* 2005;27(Special Issue 4pt2):1399–1416.

2. Finkelhor D, Hamby SL, Turner HA, Ormrod RK. *The Juvenile Victimization Questionnaire: 2nd Revision (JVQ-R2)*. Durham, NH: Crimes Against Children Research Center; 2011.

3. Hamby S, Finkelhor D, Ormrod D, Turner H. *The comprehensive JVQ administration and scoring manual*. Durham, NH: University of New Hampshire, Crimes Against Children Research Center; 2004.

4. Finkehor D, Hamby SL, Ormrod RK, Turner HA. The Juvenile Victimization Questionnaire: reliability, validity, and national norms. *Child Abuse Negl.* 2005;29:383-412.

5. Radford L, Corral S, Bradley C, Fisher H, Bassett C, Howat N, Collishaw S. *Child abuse and neglect in the UK today*. London: NSPCC; 2011.

6. Radford L, Corral S, Bradley C, Fisher HL. The prevalence and impact of child maltreatment and other types of victimisation in the UK: findings from a population survey of caregivers, children and young people and young adults. *Child Abuse Negl.* 2013;37(10):801-813.

7. Caspi A, Moffitt TE, Thornton A, et al. The life history calendar: A research and clinical assessment method for collecting retrospective event-history data. *Int J Methods Psychiatr Res.* 1996;6:101-114.

8. Bifulco A, Brown GW, Harris TO. Childhood Experience of Care and Abuse (CECA): a retrospective interview measure. *J Child Psychol Psychiatry.* 1994;35(8):1419-1435.

9. Bifulco A, Brown GW, Neubauer A, Moran P, Harris T. *Childhood Experience of Care and Abuse (CECA) training manual*. London: Royal Holloway College, University of London; 1994.

10. Bifulco A, Brown GW, Lillie A, Jarvis J. Memories of childhood neglect and abuse: Corroboration in a series of sisters. *J Child Psychol Psychiatry.* 1997;38:365-374.

11. Barnett D, Manly JT, Cicchetti D. Defining child maltreatment: The interface between policy and research. In D Cicchetti & SL Toth (Eds.), *Child* *abuse, child development, and social policy* (pp. 7-74). Norwood, NJ: Ablex; 1993.

12. Bifulco A, Brown GW, Moran P, Ball C, Campbell C. Predicting depression in women: the role of past and present vulnerability. *Psychol Med.* 1998;28:39–50.

13. Fisher HL, Bunn A, Jacobs C, Moran P, Bifulco A. Concordance between mother and offspring retrospective reports of childhood adversity. *Child Abuse Negl.* 2011;35(2):117–122.

14. March JS, Parker JD, Sullivan K, et al. The Multidimensional Anxiety Scale for Children (MASC): factor structure, reliability, and validity. *J Am Acad Child Adolesc Psychiatry.* 1997;36(4):554-565.

15. Kovacs M. *Children’s Depression Inventory (CDI) manual*. Multi-Health Systems; 1992.

16. Kim-Cohen J, Moffitt TE, Caspi A, Taylor A. Genetic and environmental processes in young children’s resilience and vulnerability to socioeconomic deprivation. *Child Dev.* 2004;75(3):651-668.

17. Poulton R, Caspi A, Moffitt TE, Cannon M, Murray R, Harrington H. Children's self-reported psychotic symptoms and adult schizophreniform disorder: A 15-year longitudinal study. *Arch Gen Psychiatry*. 2000;57:1053-1058.

18. Schreier A, Wolke D, Thomas K, Horwood J, Hollis C, Gunnell D, Lewis G, Thompson A, Zammit S, Duffy L. Prospective study of peer victimization in childhood and psychotic symptoms in a nonclinical population at age 12 years. *Arch Gen Psychiatry.* 2009;66:527-536.

19. Miller TJ, McGlashan TH, Rosen JL, Cadenhead K, Ventura J, McFarlane W, Perkins DO, Pearlson GD, Woods SW. Prodromal assessment with the structured interview for prodromal syndromes and the scale of prodromal symptoms: predictive validity, interrater reliability, and training to reliability. *Schizophr Bull.* 2003;29(4):703-715.

20. Miller TJ, Cicchetti D, Markovich PJ, McGlashan TH, Woods SW. The SIPS-Screen: a brief self-report screen to detect the schizophrenia prodrome. *Schizophr Res.* 2004;70(suppl1):78.

21. Spauwen J, Krabbendam L, Lieb R, Wittchen HU, van Os J. Does urbanicity shift the population expression of psychosis? *J Psychiatric Res.* 2004;38,:613-618.

22. Yoshizumi T, Murase S, Honjo S, Kaneko H, Murakami T. Hallucinatory experiences in a community sample of Japanese children. *J Am Acad Child Adolesc Psychiatry.* 2004;43:1030-1036.

23. Yung AR, Nelson B, Baker K, Buckby JA, Baksheev G, Cosgrave EM. Psychotic-like experiences in a community sample of adolescents: Implications for the continuum model of psychosis and prediction of schizophrenia. *Aust NZ J Psychiatry.* 2009;43:118-128.
